# Health care users’ knowledge, attitudes and perceptions of HIV self-testing at selected gateway clinics at eThekwini district, KwaZulu-Natal province, South Africa

**DOI:** 10.1080/17290376.2018.1517607

**Published:** 2018-09-03

**Authors:** Sibongiseni Daphney Gumede, Maureen Nokuthula Sibiya

**Affiliations:** a Master of Health Sciences in Nursing, Durban University of Technology, Durban, South Africa; b Faculty of Health Sciences, Durban University of Technology, Durban, South Africa

**Keywords:** HIV self-testing, oral HIV testing, South Africa

## Abstract

Progress in promoting knowledge of HIV status has been made globally, but half of all people living with HIV are still unaware of their HIV status. It is argued the new innovative HIV self-testing strategy could increase the uptake of HIV testing among the people. The aim of the study was to assess outpatients’ health care user’s knowledge, attitudes and perceptions towards HIV self-testing (HIVST) at selected Gateway clinics at eThekwini District, KwaZulu-Natal Province, South Africa. The objectives of the study were to determine health care users’ knowledge of HIVST, assess health care users’ attitudes and perceptions towards HIVST and establish if there is any relationship between knowledge, attitudes and perceptions of health care users towards HIVST. A quantitative, non-experimental descriptive design was used to determine knowledge, attitudes and perceptions of health care users at three purposefully selected Addington, R. K. Khan and Prince Mshiyeni Memorial Hospital Gateway clinics at eThekwini Health District. A convenience sampling of 442 respondents were sampled from the three study sites. Results of the study revealed that health care users had a reasonable knowledge of HIV self-testing and there were indications that they could use it if it can be made freely available to the public and be properly regulated. Generally, health care users indicated positive attitudes towards HIV self-testing. Nevertheless, issues of lack of pre and post-test counselling, false negative results and sale of unregulated testing kits seemed to be issues of concern that require addressing if HIV self-testing is to be promulgated in South Africa.

## 1. Introduction

In the year 2016, approximately 36.7 million people were living with human immunodeficiency virus (HIV) globally. Out of the 36.7 million people living with HIV, 19.4 million are living in the eastern and southern Africa which is the hardest hit by the HIV epidemic (WHO, 2017). Progress in promoting knowledge of HIV status has been made globally, but half of all people living with HIV are still unaware of their HIV status (Joint United Nations Programme on HIV/AIDS [UNAIDS], 2016). An urgent action is required to close the testing gap as 10% of young men and 15% of young women are aware of their HIV status in the eastern and southern Africa (Joint United Nations Programme on HIV/AIDS [UNAIDS], 2016). New and innovative ways to get people tested for HIV infection are needed to take HIV testing out of the clinics into the community (Department of Health, 2017). HIV self-screening test kits could increase the numbers of people that get tested and know their status (Chanda et al., 2017*;* Jamil et al., 2017; Jennings et al., 2017; WHO, 2016).

Generally, studies show the acceptability of HIVST for the general and key populations (Bustamante et al., 2016; Pant Pai et al., 2014). HIVST can be safely and accurately performed by most people. (Krause, Subklew-Sehume, Kenyon, & Colebunders, 2013). In South Africa, Mkhwanazi (2016) reported that selling of HIVST kits was for the first time officialised and are now available in 4500 South African pharmacies countrywide following the announcement by the South African Pharmacy Council (SAPC). The South African Pharmacy Council took a stance to set out minimum standards for the sale of HIV self-screening test kits from pharmacies to the public (Department of Health, 2017). Furthermore, Policy and Guidance Considerations have been formulated to up scale the safe use of HIVST as it has a potential to impact the first ‘90’ of the UNAIDS 90-90-90 targets (Southern African HIV Clinicians Society, 2017).

Currently, South Africa has made tremendous efforts to increase the number of people testing for HIV, but the uptake has not achieved the expected results (Perez et al., 2016). HIV counselling and testing campaigns have been initiated, but many people have exercised their rights to refuse to test. From the refusals to test it seems adequate testing and antiretroviral coverage will not be reached (Perez et al., 2016).

South Africa has a diverse community with potential HIV self-testers varying in literacy, comprehension and motivation levels. Potential self-testers must possess adequate knowledge of the process of self-testing and implications thereof to prevent possible abuse, psychological harm to the self-tester and other people. This study aimed at assessing knowledge, attitudes and perceptions of HIVST among health care users (HCU) in eThekwini Health District. An attitude is the way a person views something or tends to behave towards it, often in an evaluative way (Compact Oxford Dictionary Thesaurus, 2006, p. 65). A perception is an understanding or interpretation of something in a particular way (Compact Oxford Dictionary and Thesaurus, 2006, p. 669).

## 2. Methodology

The study was conducted at Addington, R. K. Khan and Prince Mshiyeni Memorial Hospitals’ Gateway clinics at eThekwini Health District, KwaZulu-Natal, South Africa. The study was a quantitative, cross-sectional descriptive design. Hospitals were purposively selected as they were believed to have population with knowledge in question which is HIVST knowledge, attitudes and perceptions. A convenience sampling of 442 respondents over the age of 18 years was arranged in the three research settings. A total of 98 respondents were selected from Addington and R.K. Khan Hospitals’ Gateway clinics respectively and 246 from Prince Mshiyeni Memorial Hospital Gateway clinic estimated daily clinic attendance numbers. The researcher did daily sampling on the available respondents meeting the criteria for inclusion in the study in each site until the total sample was reached. A statistician was consulted for advice on the sample size.

Data was collected from the 8th December 2016 to 13th January 2017 using a written survey questionnaire which consisted of 68 items, with a level of measurement at a nominal or an ordinal level. The data collection tool was in both English and isiZulu language. The tool comprised of five sections. Section one comprised personal information such as age, sex, gender, language, educational standard, religion and residence. Section two comprised health care users’ knowledge of HIVST. Section three comprised assessment of attitudes of respondents towards HIVST and linkage to care following HIVST. Section four comprised attitudes of health care users towards HIV status disclosure should the test be positive. The last section assessed perceptions of health care users towards HIVST.

Data collection commenced after the researcher was granted full ethical clearance from the Institutional Research Ethics Committee (Ethics Number 109/2016) and permission to conduct the study was received from the Provincial Department of Health, eThekwini District Health Office and the Chief Executive Officers of hospitals where the selected Gateway clinics are situated.

The data collected was analysed using SPSS version 23.0. Tests that were used in data analysis were descriptive statistics including means and standard deviations where applicable.

## 3. Results

### 3.1. Personal information

There were 442 HCU who completed the questionnaires. Personal information is illustrated in Table 1. The majority of the HCU were females (68.8%), mostly were between the ages 18–29 years (42.5%). IsiZulu home language was mostly spoken by HCU (73.3%) followed by English home language (13.1%). Most of HCU belonged to the Christian religion (76.5%) with some 60.2% having some or all high school education. They were mostly single (62.2%), residing in townships (46.4%) and urban areas (27.4%) respectively. Most of them were working (41.6%) and those that were unemployed were (39.6%).

**Table 1.** Demographic information of respondents *n* = 442.

*Gender*	* *
Male	30.1%
Female	68.8%
Unspecified	1.1%
*Age*	* *
18–29	42.5%
30–39	22.9%
40–49	15.2%
50–59	6.1%
60+	4.1%
Unspecified	9.3%
*Language*	* *
IsiZulu	73.3%
English	13.1%
isiXhosa	8.8*
Other	4.8%
*Religion*	* *
Christian	76.5%
Hindu	4.1%
Muslim	3.8%
Nazareth	13.3%
Other	3.7%
*Education*	* *
Some/all primary school	10.0%
Some/all high school	60.2%
Tertiary education	29.6%
Missing system	.2%
*Occupation*	* *
At school	7.5%
Student	18.8%
Working	41.6%
Unemployed	39.6%
*Marital status*	* *
Single	62.2%
Partnered	12.4%
Widowed	6.1%
Married	16.5%
Divorce/separated	2.7%
*Residence*	* *
Rural	14.0%
Urban	27.4%
Township	46.4%
Informal settlement	8.4%
Peri-urban	3.8%

### 3.2. HCU knowledge of HIVST

Table 2 illustrates HCU knowledge of HIVST. Results of the study revealed that the majority of HCU (69.9%) had heard about HIVST. Knowledge was also possessed through HCU having read about HIVST (42.8%). Few HCU (23.5%) had seen and (18.8%) had used an HIVST. Knowledge on the availability and use of HIVST was also determined. Some HCU (41%) had knowledge that HIVST kits are available from private pharmacies whilst only (31.2%) had knowledge that HIVST was available from the internet. Most HCU indicated that HIVST is done using blood (63.8) with less indicating that fluid from the mouth can also be used (18.1%). More females (56.25%) possessed more knowledge of HIVST than males (43.75%).

**Table 2.** Health care users’ knowledge of HIVST *n* = 442.

									Yes	No	Not sure
I have heard about HIVST									69.5%	25.1%	5.4%
I have seen an HIVST									23.5%	65.6%	10.2%
I have used an HIVST									18.8%	73.3%	7.7%
I have read about HIVST									42.8%	49.8%	7.5%
It is legal to use HIVST kits in South Africa									35.3%	29.4%	35.3%
HIVST are available from private pharmacies									41.0%	20.1%	38.2%
HIVST kits are available in government clinics/hospitals									30.8%	31.9%	37.1%
HIVST kits are available on the internet									31.2%	23.3%	45.0%
HIVST is done using blood									63.8%	9.3%	26.7%
HIVST is done using fluid from the mouth									18.1%	40.7%	41.2%
A person can perform the HIVST on herself/himself									68.1%	17.2%	14.7%
It takes 20–40 minutes to get results from the HIVST									26.0%	17.0%	57.0%
The test can be negative if the HIV infection is less than three months									37.3%	17.0%	44.8%
A person needs to re-test after three months if the test is negative									71.9%	9.5%	18.1%
There is a telephone hotline to call should the test be positive									31.0%	19.9%	48.9%
A person needs to be counselled by the HIV counsellor before taking the HIV self-test									66.1%	17.4%	16.3%

### 3.3. Attitudes of HCU towards HIVST and linkage to care

A Likert scale with questions was used to determine agreements or disagreements on given attitudes towards HIVST and linkage to care. Generally, HCU indicated positive attitudes towards HIVST and linkage to care. HIVST was seen as an acceptable idea as HCU (66%) indicated. Majority HCU (69.2%) indicated that they could be able to perform an HIVST at home. Most HCU (65.6) would prefer to self-test alone with a slightly lesser number (59.3%) preferring to self-test with a partner. There were also more agreements to linkage to care as 83.9% indicated that follow up an HIV positive result at the clinic was important. Majority of respondents (82.5%) indicated that it is important to get counselling after the HIV positive test result. Attitudes towards HIVST and linkage to care is illustrated in Table 3.

**Table 3.** Attitudes towards HIVST and linkage to care *n* = 442.

	Yes	No
HIVST is a good idea	66%	40.0%
I can be able to do an HIVST at home	69.2%	22.4%
I think I would find the HIV self-testing procedure difficult to perform	25.3%	54.3%
I would prefer to self-test alone	65.6%	23.9%
I would prefer to self-test at the health facility	46.4%	34.4%
I would prefer to self-test with partner	59.3%	26.5%
I would prefer to self-test and read the results myself	67.2%	19.9%
I would like to get telephone counselling before the HIVST	53.6%	28.7%
I would seek help from the clinic should the test be positive	79.9%	9.7%
I would like to get face to face counselling after the test	77.4%	10.4%
It is important to follow up an HIV positive result at the clinic	83.9%	5.9%
It is important to get counselling after the test	82.5%	7.7%

### 3.4. Attitudes of HCU towards HIV positive status disclosure

There was reasonable agreement from respondents that an HIV test should be a total secret (*M* = 3.28, SD = 1.225), *t* (442) = 4.437, *p* < .0005 and that an HIV positive person should tell their sex partner only (*M* = 3.31, SD = 1.329), *t* (441) = 5.325, *p* < .0005. There was strong agreement that HIV positive people should inform all significant others (*M* = 3.31, SD = 1.202), *t* (440) = 5.197, *p* < .0005 and people should talk openly about it (*M* = 3.64, SD = 1.088), *t* (442) = 12.375, *p* < .0005. Attitudes towards HIV positive status and linkage to care are illustrated in Table 4.

**Table 4.** Attitudes and perceptions of health care users towards HIV self-testing and HIV positive status disclosure.

	Yes
HIV positive test should be a total secret	53.4%
HIV positive people should tell their sex partner only	55.9%
HIV positive people should inform all significant others	55.9%
HIV positive people should talk openly about it	67.2%
Privacy is ensured	66.8%
Less time is spent in clinics and hospitals	65.6%
More people can know their status	81.2%
People who are scared to go to the clinics can test at home	77.1%
People can get ARVs before they can get sicker	74.9%
There could be less transmission of HIV to other people	77.4%
People could be tested more frequently	80.8%
People could read/interpret results incorrectly	61.5%
People may not be able to read instructions properly	62.5%
People could intentionally infect others if not properly counselled before the test	44.2%
Children and workers could be tested against their will	54.6%
Family members could be tested against their will, which could result in abuse	50.0%
People could blame others should they test positive	44.2%
Should a person who has not received counselling test positive, he/she may commit suicide	63.8%
If not properly regulated by government, unreliable test kits could be sold thus giving wrong results	67.2%

### 3.5. Perceptions of health care users towards HIVST

Most HCU perceived HIVST as a strategy that could make more people to know their HIV status (81.2%) and they can also test more frequently (80.8%). It was also perceived as a strategy that there could lessen HIV transmission amongst people (77.4%). It could help people who are scared to go to the clinics to test at home (77.1). The majority of HCU (65.6%) indicated that privacy could be ensured through the use of HIVST. More than half HCU (63.8%) reported that a person may commit suicide if he has not received counselling and tests HIV positive. Unreliable HIVST kits could be sold which could give wrong results if the government does not regulate sale of HIVST (67.2%). Majority of HCU indicated that people may not be able to read instructions properly (62.5%) whilst (61.5%) indicated that people could read/interpret results incorrectly. Just more than half of the respondents indicated that there could be testing of children and workers (54.6%) against their will whilst and family members (50%) could also be tested against their will which could result to abuse. Perceptions of HCU are illustrated in Table 4.

## 4. Discussion

The study assessed knowledge, attitudes and perceptions of HCU towards HIVST as an alternative and innovative strategy of HIV testing at eThekwini district, KwaZulu-Natal, South Africa. The present study unveiled four important findings. Firstly, HCU indicated knowledge of HIVST as they have heard or read about it. Secondly, HCU had positive attitudes towards HIVST as they reported it as a good idea. Indications were that they would use it if it is regulated and officially promulgated. Thirdly, HIVST was perceived as potentially beneficial as it can provide privacy and thus more people can test and be initiated on ARV’s if tested positive. Lastly, there were perceived trepidations about the possible abuse of the HIVST kits to vulnerable groups of people such as children and women, especially if the government does not regulate the use thereof.

Personal information in the study revealed that more females (68.8%) were respondents in the study. Women make more health facility contacts for reproductive health, well baby care, minor ailments, etc. Health care services are believed not to be male user-friendly, hence males may benefit from HIVST (Makusha et al., 2015; WHO, 2016). The isiZulu language was dominant in the study as HCU (73.3%) indicated that they spoke isiZulu. According to Statistics South Africa 11 519 234 (77.8%) people speak isiZulu in the KZN province. Most HCU (60.2%) had some or all high school education which could enable them to be able to follow the instructions to perform the HIVST. According to Pant Pai et al. (2013), this level of education should be sufficient to facilitate the performance of HIVST (Pant Pai et al., 2013).

Knowledge of HIVST was established as most HCU (69.9%) had heard and 42.8% had read about HIVST (Heard & Brown, 2016; Mokgatle & Madiba, 2017; Pal et al., 2016). Conversely, the use thereof was minimal (Prestage et al., 2016) and HCU (41%) did not know that HIVST kits are available from private pharmacies and not from government institutions (Estem, Catania, & Klausner, 2016; Witzel, Rodger, Burns, Rhodes, & Weatherburn, 2016). Greacen et al. (2016) recommended that HIVST kits should be available for free in health care centres especially for the key populations.

Although HCU possessed knowledge of HIVST as they have heard or read about it, they did not possess adequate knowledge of the procedure of carrying out the testing procedure as only 26% of HCU in this study knew that it takes about 20–40 minutes to get results from the HIVST (Mokgatle & Madiba, 2017). Furthermore, very few HCU (37.3%) knew that HIVST results can be negative if the infection is less than three months old (FDA, 2014; Peate, 2015; Van Dyk, 2013; WHO, 2015). However, most HCU (71.9%) knew that re-testing after three months is necessary if the test is negative. The WHO Testing Guidelines state that re-testing is desirable only for HIV negative persons who report recent or continuing risk of exposure (WHO, 2015). Studies have documented the need for follow-up care with health care provider if the HIV test is positive and HCU (74.4%) demonstrated that they know that follow up with the health care provider is essential if HIVST is positive (Figueroa, Johnson, Verster, & Baggaley, 2015; Makusha et al., 2015; WHO, 2015).

It has been documented in various studies that HIVST is acceptable in both key and general populations and results of this study were no different. More than half HCU (66%) felt that HIVST was a good idea (Heard & Brown, 2016; Marlin et al., 2014; Mavedzenge, Baggaley, & Corbett, 2013; Pant Pai et al., 2013; Perez et al., 2016). Reasons such as confidentiality, convenience, fear of stigma, and privacy, have been cited as reasons for acceptability of HIVST (Brown, Carballo-Diéguez, John, and Schnall (2016; Ch & Young, 2016; Mokgatle & Madiba, 2017). Due to its acceptability, few HCU (25.3%) indicated that they would find the HIVST procedure difficult to perform (Marley et al., 2014). More research is needed to determine the feasibility and acceptability of HIV self-testing kits (Krause et al., 2013).

Most HCU (65.6%) indicated that they would prefer to test alone (Young et al., 2014). There was a unanimous indication that HCU (83.9%) would seek help and follow up an HIV positive result with a health care worker at the clinic should the test be positive (Cherutich, Kurth, Musyoki, Kilonzo, & Maina, 2014; Makusha et al., 2015; Ng et al., 2012; Pant Pai et al., 2013). However, Peate (2015) lamented that HIVST lacks linkage to care for people who test HIV positive as seeking help from a health worker is an individual responsibility. Confidentiality has been cited above as one of the reasons for acceptance of HIVST and just over half (53.4%) of respondents would keep an HIV positive test result confidential, although disclosure by the individual to a sexual partner, family member, trusted others or health care providers may be highly beneficial with considerable benefits (Strauss, Rhodes, & George, 2015; WHO, 2015). HCU (66.8%) do not trust health care workers to maintain confidentiality and privacy (Heard & Brown, 2016; Madiba, Segobola, & Mokgatle, 2015; Mugo et al., 2017; Nkuna & Nyazema, 2016; Pal et al., 2016; Perez et al., 2016; Rosengren et al., 2016).

Majority of HCU (81.2%) reported that HIVST could assist in the uptake of HIV testing in both general and key populations (Bavinton et al., 2013; Brown, Folayan, Imosili, Durueke, & Amuamuziam, 2014; Han et al., 2014; Van Dyk, 2013; Wood, Ballenger, & Stekler, 2014). The increased uptake of HIV testing could lead to more people getting ARVs before becoming sicker (Jahanbakhsh, Mostafavi, & Haghdoost, 2015; Nkuna & Nyazema, 2016; WHO, 2016). Additionally, HCU (80.8%) indicated that the frequency of testing among people could be increased if HIVST can be legally available in the country (Brown et al., 2016; Prestage et al., 2016; Yan et al., 2015).

Even though HIVST was viewed as a good idea as indicated above, most HCU (61.5%) reported fears that people could read or interpret results incorrectly. De La Fuente et al. (2012) established misinterpretation of HIVST results especially among people older than 30 years of age, Latin Americans, and those without university education. A concern was likewise indicated as HCU (66.1%) reported that people may not able to read and understand instructions properly (Choko et al., 2015; Ng et al., 2012; Pant Pai et al., 2013; Perez et al., 2016; Sarkar et al*.*, 2016).

There were no documented serious unintended consequences found that were related to HIVST such as intentional infection of other people related to not getting pre-test counselling (Choko et al., 2015), except for a study by Heard and Brown (2016) in which one participant mentioned he would intentionally infect others. In this study HCU (67.4%) reported that a possibility exists that an HIV positive person could intentionally infect others if not properly counselled before HIVST (Makusha et al., 2015; Perez et al., 2016; Van Dyk, 2013).

There was also reported apprehensions from more than just half HCU (54.6%) that children, workers and family members could be tested against their will, resulting in abuse (Makusha et al., 2015; Perez et al., 2016). Nonetheless, there was no data to support this claim in the South African context (Makusha et al., 2015). HCU (63.8%) reported another possible shortcoming of HIVST that people may also try to commit suicide if not properly counselled before the HIVST (Choko et al., 2015; Makusha et al., 2015; Ng et al., 2012; Pal et al., 2016).

In South Africa, HIVST has not been regulated for sale either online or from the private pharmacies and HCU (67.2%) reported that if HIVST is not properly regulated by the government, unreliable test kits could be sold to people which could give wrong results. In countries where HIVST trade is unregulated, a possibility of illegal selling of unreliable test kits exists (Koutentakis et al., 2016; Williams, Dean, Harting, Bath, & Gilks, 2016).

## 5. Recommendations

Information on HIVST should be disseminated to people so that possible abuse can be averted as these are available even if they are not legalised. Awareness of HIVST can be made possible through the use of media, newspapers and government institutions.

Exploration of the use of HIVST should include ethical and legal controls, innovative methods of pre- and post-test counselling and linkage to care as these have been identified as needs surrounding HIVST in the study. Stakeholders need to explore the feasibility of incorporating HIVST into health care services especially for those who refuse conventional testing methods. More information should be cascaded to people so that they can make informed decisions and be protected from potential abuse from HIVST kits which are available in pharmacies countrywide.

## 6. Conclusion

The study results indicated that majority of the respondents demonstrated knowledge of HIVST through having heard about it. HIVST was seen as a good idea and respondents did not perceive that they would have any difficulty to perform it. Positive aspects of HIVST identified in the study were that privacy could be ensured and it could be a strategy to prevent onward HIV transmission. However, there were also concerns raised, such as the possibility of coerced testing and abuse from the use of HIVST, especially with women and children.

## 7. Strengths and limitations of the study

One of the significant findings of this study was that despite the fact that HIVST is not legalised in South Africa, respondents have heard about it even though they have never seen or used it. Some HCU have used HIVST which further emphasises the need for consideration of regulating the use or non-use thereof. It was also important to note it was seen as a good idea which could provide one of the options for HIV testing for those who do not want to use the traditional approaches to testing. The use of HIVST can ensure privacy and increase the uptake of HIV testing, however, there were concerns that if not properly regulated, violence and abuse of women and children can occur.

The study had some limitations. The study was conducted in Gateway clinics in hospitals around Durban which have basically homogenous characteristics as the institutions are in urban, peri-urban areas and townships, so this study cannot be generalised to other settings. Secondly, the study was cross sectional and data was collected over a short period of time from the 8th December 2016 to the 13th January 2017.
